# Prenatal Origin of Childhood Overweight, Obesity and Insulin Resistance, with Special Emphasis on Maternal Diabetes, Excessive Weight, Nutrition and Hormone Imbalances

**DOI:** 10.3390/nu18142330

**Published:** 2026-07-16

**Authors:** Asher Ornoy, Boniface Echefu, Maria Becker

**Affiliations:** 1 Department of Morphological Sciences and Teratology, Adelson School of Medicine, Ariel University, Ariel 40700, Israel; bonifacee@ariel.ac.il (B.E.); mariabe@ariel.ac.il (M.B.); 2 Department of Medical Neurobiology, Hebrew University Hadassah Medical School, Jerusalem 91120, Israel

**Keywords:** prenatal origin, maternal overweight, childhood obesity, insulin resistance, metabolic syndrome, diabetes, epigenetic effects

## Abstract

Overweight in childhood, adolescence and adulthood is now a world-wide epidemic. A variety of preventive and therapeutic measures are being developed with partial success. Since the pioneering studies of D.J. Barker linking maternal famine in pregnancy to long—term undesirable effects on the offspring—the “metabolic syndrome” that stems from long-lasting epigenetic changes, it became evident that there are many undesirable events which affect in-utero fetal growth and development. They often persist postnatally, through childhood and adulthood. The purpose of this review is to discuss the prenatal factors that may have long term effects on postnatal weight gain and obesity that are important contributors to the “obesity” epidemic. These prenatal undesired factors are pregestational and gestational diabetes, pre-pregnancy maternal obesity, excessive weight gain in pregnancy, placental dysfunction and maternal hormonal imbalances. They may affect fetal growth, generally causing excessive growth (macrosomia) and sometimes diminished growth (small for gestational age). These prenatal conditions are associated with epigenetic changes that may contribute to insulin resistance and subsequent metabolic alterations leading to increased food intake, overweight, and obesity. Studies also demonstrate that increased exposure in pregnancy to teratogens, like endocrine disruptors and cigarette smoking, may also affect prenatal and postnatal growth and adiposity. However, many the studies demonstrating the role of these factors are often weakened, as they mainly demonstrate associations and do not sufficiently consider possible confounding factors such as postnatal age, nutrition and lifestyle. Better understanding of these prenatal factors may support more effective prevention and management strategies for childhood overweight and obesity.

## 1. Introduction

Prenatal and postnatal growth are influenced by genetic and environmental factors. Among the non-genetic environmental factors affecting the developing embryo and fetus are maternal over- or undernutrition, maternal obesity, excessive gestational weight gain, and related metabolic disturbances. They are often linked to fetal overgrowth and increased postnatal adiposity [1,2,3,4,5]. Disturbed placental function is an additional important factor [2,3]. These changes in intrauterine growth patterns may subside after birth but often have a major effect on postnatal growth, increasing weight gain and adiposity. Moreover, even infants with reduced growth in utero may become overweight after birth [2]. Well-studied examples are maternal diabetes in pregnancy that induces in humans and in experimental animals a variety of epigenetic changes with long-term effects on postnatal growth and health in childhood and adulthood [6,7,8].

Only about 80% of newborn infants have birth weight in the normal range, as about 10% are born small for gestational age (SGA) and 5–10% have excessive weight at birth (macrosomia) [9]. SGA and macrosomia may be associated with preterm or post-term delivery [10,11], congenital anomalies, and neurodevelopmental alterations, and often have long-term neurodevelopmental consequences [6,11,12,13]. Abnormal fetal growth may also induce insulin resistance, type II diabetes, obesity, dyslipidemia, hypertension, and cardiovascular diseases in childhood, adolescence, and adulthood [6,14,15]. Finally, maternal exposure to teratogens (i.e., maternal infections, maternal alcohol abuse, heavy smoking) may affect intrauterine growth that also persists postnatally [3,16].

According to the Developmental Origins of Health and Disease (DOHaD) hypothesis [17], inadequate nutrient availability during fetal life may be associated with epigenetic changes that contribute to adaptive metabolic programming, commonly referred to as the “thrifty phenotype” or “metabolic syndrome” [18]. This adaptive response enhances energy efficiency and prioritizes survival under conditions of intrauterine scarcity. However, when individuals exposed to such fetal undernutrition encounter an energy-rich postnatal environment, this metabolic programming may become maladaptive, increasing the risk of obesity, insulin resistance, and cardiometabolic diseases later in life [18].

Animal models are often used to support or antagonize clinical phenomena. However, they can provide biological explanations for human diseases and help to explore treatment modalities, but do not, on their own, validate findings in human diseases. Indeed, many animal models using protein deficiency diets, low- or high-calorie diets, or other dietary manipulations during pregnancy have been published. They generally approved many human findings related to the DOHaD hypothesis and outlined the molecular phenomena of these epigenetic changes [19].

Since the metabolic syndrome (DOHaD) is a predominant cause of childhood overweight, obesity, and serious complications in adulthood [20], many studies have delineated possible ways to alleviate or prevent, in adulthood, some of the long-term consequences of the metabolic syndrome [21,22,23,24]. However, as this is not the aim of this review, we will discuss these issues only briefly.

Many prenatally originating effects are epigenetic in nature, inducing long-term changes in the expression of different genes related to energy metabolism and/or to growth factors. Well-defined prenatal causes influencing postnatal weight and health will be discussed in this review, especially the role of maternal nutrition, exposure to noxious environmental agents, and hormone imbalances [2,18,21,22]. Finally, insulin resistance seems to be a very important contributor to different health complications related to the long-term effects of negative changes in prenatal metabolic development.

We believe that this group of obese children needs a comprehensive clinical approach, identifying the possible prenatal factors leading to childhood obesity. Proper assessment may contribute to improved understanding and potentially improve preventive strategies.

For this narrative review, we conducted a literature search in PubMed/Medline, Scopus, and Google Scholar for studies published between 2000 and 2026. Search terms included combinations of “maternal obesity”, “gestational diabetes”, “diabetic pregnancies”, “fetal programming”, “DOHaD”, “fetal growth”, “maternal metabolic hormones”, “placental endocrine signaling”, “fetal adaptive responses”, “insulin resistance”, “epigenetics” and “postnatal growth”, “offspring adiposity” and “offspring metabolic outcomes”. Priority was given to large cohort studies, systematic reviews, and meta-analyses. Additional relevant articles were identified through reference screening. Studies were selected based on relevance to prenatal influences on postnatal overweight/obesity or metabolic outcomes. Studies were included only if they described the effects of prenatal factors on postnatal overweight and obesity. Special emphasis was given to studies that discuss the epigenetic changes that are associated with DOHaD. Studies were excluded if they lacked relevance to prenatal exposures or did not report postnatal metabolic outcomes. As this is a narrative and not a systematic review, we applied a structured search strategy but did not apply defined inclusion criteria and quality assessment.

## 2. Maternal Diseases That Affect Prenatal Growth and Cause Postnatal Obesity

### 2.1. Maternal Diabetes in Pregnancy

Pregestational diabetes mellitus (PGDM) may be associated with an increased rate of spontaneous abortions, intrauterine death, and congenital malformations among the offspring [15]. Gestational diabetes mellitus (GDM) does not seem to increase the rate of congenital malformations in the offspring but might affect fetal growth and be associated with a higher rate of health problems later in life, including overweight and obesity or neurobehavioral problems, which may start in childhood. The prevalence of these complications is reduced by optimal glycemic control. Offspring of diabetic mothers may be macrosomic, SGA, or of normal birth weight, depending on the severity of diabetes, presence or absence of complications, and the degree of diabetic control [6,15].

Maternal hyperglycemia increases transplacental glucose delivery, stimulates fetal pancreatic insulin secretion, leading to enhanced lipogenesis, glycogen storage, and somatic growth [15,23]. Fetal hyperinsulinemia results in overgrowth, whereas fetal insulin deficiency is associated with intrauterine growth retardation (IUGR) [24,25]. Optimal control would normalize fetal growth, while poorly controlled diabetes or diabetic complications (i.e., diabetic nephropathy) often result in small or large-for-gestational age infants [26,27].

Several studies have examined the maternal insulin growth factor 1 (IGF-I) and insulin growth factor binding protein 1 (IGF1BP) system in pregnancies complicated by diabetes. In diabetic pregnancies, maternal circulating IGF-I levels are generally reduced, whereas fetal IGF-I concentrations are increased in infants born to diabetic mothers compared with non-diabetic controls [28,29]. Elevated IGFBP-3 levels have also been reported in both maternal and fetal serum in T1DM pregnancies [30]. Disruption of the placental growth hormone (PGH)–IGF-I–IGFBP-3 axis in T1DM pregnancies may alter normal fetal growth regulation and contribute to fetal overgrowth and macrosomia [30].

Various studies reported the postnatal growth in children of diabetic mothers. Generally, they found increased weight, especially during preadolescence and adolescence [31,32,33,34,35,36,37]. Most studies reported that maternal diabetes is associated with overweight and obese children, especially if they were born macrosomic [32,35,36,37]. These associations were more pronounced in offspring of diabetic mothers with T1DM, mothers with high BMI, and mothers with high fasting glucose blood levels [36]. Macrosomic children born to diabetic mothers, but not macrosomic infants born to non-diabetic mothers, were also at high risk of developing the “Metabolic Syndrome” with insulin resistance [32].

We examined 5–12-year-old children born to mothers who developed GDM and to mothers with well-controlled PGDM in comparison to children born to healthy control mothers [33,34]. While we found no difference in cognitive ability, head circumference, or height between the children of diabetic mothers and controls, they exhibited a significant increase in body weight and BMI. The differences were more pronounced in the elder children aged 9–12 (preadolescence) compared to the younger, 5–8-year-old children.

**In summary**, multiple cohort studies and meta-analyses consistently demonstrate an association between maternal diabetes and increased offspring adiposity, particularly during adolescence. Outcomes vary depending on glycemic control, type of diabetes and severity, and maternal metabolic status. The strength of these associations is often reduced after adjustment for maternal pre-pregnancy BMI, highlighting the complexity of causal interpretation. Moreover, the possible effects of postnatal nutrition on the child’s health and disease are generally not considered. Therefore, causal interpretations should be made with caution. Optimal glycemic control in pregnancy may reduce the risk of most long-term effects of diabetes on postnatal overweight and metabolic consequences.

### 2.2. The Effects of Maternal Overweight in Pregnancy on Growth During Childhood

Maternal obesity is frequently accompanied by hyperinsulinemia and increased insulin resistance, expanded pancreatic beta-cell mass, and high insulin secretion, further stimulating placental nutrient transport, interacting with the IGF signaling pathway to promote fetal tissue growth [38]. Various studies support the importance of the placental growth factor (PGH) and IGF axis in determining fetal growth outcomes, and maternal plasma PGH levels were positively associated with gestational age and fetal growth [39,40].

There is a direct relationship between maternal obesity and fetal and postnatal growth [41,42,43,44,45]. Excessive gestational weight has been linked to a higher risk of adverse pregnancy outcomes, including hypertensive disorder of pregnancy, gestational diabetes, Cesarean delivery, preterm birth, and the delivery of macrosomic infants [45]. These macrosomic infants tended to develop adulthood type 2 diabetes, hypertension, and a higher mortality rate from coronary artery disease compared to normal-weight newborns of obese mothers. A high maternal pre-pregnancy body mass index (BMI) increased the risk of preeclampsia [46] and of developing gestational diabetes [47]. Khashan et al. carried out a population register-based cohort study from the North Western Perinatal survey (*n* = 99,403 babies born during 2004–2006) and found that overweight and obese women have a higher risk of macrosomia and Cesarean delivery and a lower risk of preterm delivery [43]. The relative risk of macrosomia in relation to being overweight, obesity, and morbid obesity increased by 1.7, 2.7, and 4.8, respectively.

Offspring of overweight women are at high risk of developing overweight and abdominal obesity, i.e., disproportionate accumulation of abdominal fat that predisposes to a higher risk of cardiovascular disease [48,49]. In a cohort of 4168 children from the longitudinal Northern Finland cohort of 1986, the rate of overweight in the offspring of mothers who had both GDM and pre-pregnancy overweight was 40% (OR of 4.05), and abdominal obesity was observed in 25.7%. In non-diabetic obese women, it was reduced to 27.9% and 19.5%, respectively [49].

Maternal overnutrition in pregnancy may induce early hyperinsulinemia in the offspring, promoting subsequent adiposity, insulin resistance, and emerging dysglycemia, supporting an insulin-hypersecretion pathway possibly leading to insulin resistance [50].

While it is accepted that maternal overweight and obesity may be associated with overweight/obese children, there are other factors that may modify this association and are not often considered. For example, a Cesarean section that was associated with childhood overweight/obesity and the duration of breastfeeding may modify the association of maternal overweight with weight at childhood [51].

Several investigators described an association of maternal obesity with a higher risk of fetal growth restriction (SGA) [52,53,54]. SGA infants were seen mainly among women whose gestational weight gain was within the recommended range. However, these SGA infants also tend to become overweight and obese [2].

### 2.3. The Effects of Excessive Weight Gain in Pregnancy and Postnatal Growth

The optimal increase in maternal weight during pregnancy is largely dependent on the maternal BMI before pregnancy. The American Society of Obstetrics and Gynecology recommends optimal weight gain in relation to pre-pregnancy BMI: for underweight, low BMI women, 12.7–18.1 kg, for women with normal body weight, 11.3–15.9 kg, and for overweight and obese women (BMI over 30), it should be no more than 6.8–11.3 kg [55]. Accordingly, pregnant women in any of these categories who gain more weight may endanger themselves and their children.

Several investigators assessed the association between maternal gestational weight gain (GWG), pre-pregnancy body mass index (ppBMI), and offspring adiposity [56,57,58]. Mothers of children who were overweight/obese at the time of study generally gained more weight during pregnancy compared to mothers of children who had a normal weight. Maternal age at delivery did not affect the child’s weight [59]. Positive associations of GWG and ppBMI with offspring overall adiposity were found. Higher maternal ppBMI was directly related to higher offspring leptin, High-sensitivity C-reactive protein (hsCRP), IL-6, high systolic blood pressure (BP), and low adiponectin [56] whereas GWG was associated with low leptin levels. Greater maternal pre-pregnancy weight and GWG up to 36 weeks of gestation were associated with offspring adiposity and adverse cardiovascular risk factors [57]. Large-scale pooled evidence with long-term follow-up concluded [56] that higher maternal ppBMI and gestational weight gain were associated with increased risk of childhood overweight/obesity.

**In summary**, there is plenty of data showing that both pre-pregnancy high BMI and high weight gain in pregnancy are associated with long-term increase in postnatal weight and may induce several important metabolic and endocrine changes in the offspring, which lead to postnatal overweight and obesity. These data are weakened by various confounding factors in pregnancy and postnatally, such as maternal hypertension, placental dysfunction, and postnatal nutrition of the child, as they were seldom assessed. Neither the possible genetic differences nor other negative events in pregnancy were taken into consideration.

## 3. Insulin Resistance as a Major Mechanism of the Long-Term Effects of Maternal Diabetes, Overweight or Underweight on Offspring Adiposity

Several hypotheses have been proposed to explain the observed effects in offspring of diabetic or overweight mothers, among which epigenetically mediated insulin resistance represents a central and extensively studied mechanism. Other possible mechanisms are changes in leptin secretion, leptin insensitivity, and hypothalamic programming.

### 3.1. The Role of Insulin and Insulin Resistance

Fetal insulin is one of the most important anabolic hormones regulating intrauterine growth [7,60,61]. Maternal insulin does not cross the placenta; therefore, fetal insulin secretion is directly driven by maternal glucose availability [62,63,64]. Partial maternal insulin resistance develops in pregnancy from the end of the first trimester as a physiological adaptation evoked by placental hormones and maternal adipokines [65,66]. Maternal insulin resistance is associated with increased fetal fat deposition [67].

Insulin resistance syndrome seems to be the fundamental underlying problem in the pathogenesis of the “metabolic syndrome” (syndrome X, thrifty hypothesis, DOHaD) [68]. This term refers to the constellation of obesity, hyperglycemia, hyperlipidemia, and hypertension that cluster together to form a syndrome with possible severe health consequences [68]. The maternal and fetal nutritional imbalance during pregnancy induces over-secretion of insulin that may cause insulin resistance in the fetus. Insulin resistance causes insulin over-secretion followed by insulin deficiency, a very important etiological factor in the later development of type 2 diabetes and cardiovascular disease [64,65,66,67].

Insulin is an important mediator of hypothalamic circuits. Elevated fetal insulin induced by maternal diabetes may affect the hypothalamic development [67,68,69,70,71,72,73,74]. Insulin in the brain decreases food intake, while insulin depletion (or resistance) may promote hyperphagia. Singh et al. found a decrease in Neuropeptide Y (NPY), a protein that increases food intake, in the rat fetal hypothalamus when the dams were made diabetic by streptozotocin injection [74]. Fetal intracerebral injection of insulin with normal blood glucose levels caused a decline in the NPY protein, suggesting that insulin directly reduces NPY levels in the brain and, as a result, reduces food intake; insulin resistance may, therefore, increase food intake.

Gestational undernutrition, typically expressed as fetal growth restriction (SGA), follows a parallel, yet distinct pathway toward insulin resistance. Long-term follow-up of SGA infants reveals increased adiposity and insulin resistance, particularly when rapid catch-up growth occurs, aligning with the thrifty phenotype model wherein metabolic adaptations to scarcity become maladaptive in postnatal abundance [2,6,75]. Studies on DOHaD demonstrated that lower birth weight predicts adult insulin resistance and metabolic syndrome, features independent of adult BMI, affirming long-term consequences of early nutrient deprivation [18,76]. Thus, both undernutrition and overnutrition increase the risk of later metabolic disorders, although the strength and direction of these associations vary across populations and study designs [77].

Together, these findings show that opposite forms of gestational malnutrition ultimately converge on the same metabolic trajectory: programmed insulin resistance emerging in childhood and worsening through adolescence. Nutrient excess drives this pathway via fetal hyperinsulinemia, enhanced adipogenesis, and early adiposity rebound, whereas nutrient deprivation triggers the classic thrifty phenotype-reduced fetal growth, impaired glucose oxidation, and lower insulin secretion-reflecting a conservative metabolic adaptation to constraint [6]. These converging observations demonstrate how metabolic adaptations shaped by either constraint or excess become maladaptive when postnatal conditions diverge from fetal expectations, ultimately yielding a unified, developmentally programmed insulin-resistant phenotype [6,18,32,36].

### 3.2. Interventional Methods to Alleviate the Long-Term Consequences of the Metabolic Syndrome

Insulin resistance seems to be very common and is apparently the most common non-communicable disease [20,78,79]. Most cases of insulin resistance do not stem from events in pregnancy, but they have similar long-term outcomes: cardiovascular disease (hypertension, hyperlipidemia, and overweight/obesity), hyperglycemia, and type 2 diabetes [78,79].

Moreover, the long-term consequences do not seem to be dependent on etiology and are therefore similar. Hence, the same preventive and therapeutic measures are relevant for all cases, regardless of etiology.

The preventive and therapeutic measures include the use of Phytochemicals and vitamin D [80], physical activity and weight loss, statins and hypotensive drugs [81,82], polyphenols [83], antioxidants and pharmacological agents such as melatonin, resveratrol and probiotics/prebiotics, and drugs that control blood pressure [20]. None of these measures was shown to change the basic molecular and metabolic changes induced by insulin resistance.

**In summary**, due to the crucial role of insulin in many metabolic pathways and in different organs, insulin resistance originating from prenatal events and leading to insulin depletion seems to be the most important mechanism underlying the development of overweight and obesity. In addition, it may cause significant damage to many organs, inducing hyperlipidemia, hypertension, diabetes, and cardiovascular disease. For additional information, see Section 5.6.

## 4. The Role of Hormones in Fetal Growth and the Effects on Postnatal Weight

Multiple endocrine and metabolic adaptations take place during pregnancy to secure sufficient energy, hormones, and nutrient supply to the fetus [84,85]. Adipokines, particularly leptin and adiponectin, are key clinical biomarkers of maternal metabolic adaptation in pregnancy and are linked to fetal growth and early postnatal body composition. Similarly, growth hormone–insulin-like growth factor (GH- -IGF) plays a major role in prenatal and postnatal growth.

### 4.1. Leptin

Maternal leptin concentrations increase during pregnancy in proportion to adiposity and reflect the degree of pregnancy-associated insulin resistance. Elevated leptin levels in early pregnancy have been shown to predict the subsequent development of GDM [86], and correlate strongly with maternal anthropometric measures and fat mass [87].

During pregnancy, leptin is produced by maternal adipose tissue and by the placenta, particularly by syncytiotrophoblastic cells [88,89,90]. Leptin levels, especially hyperleptinemia, and leptin resistance, contribute to changes in energy balance during pregnancy and are associated with excessive gestational weight gain [91,92,93].

The maternal plasma leptin levels increase progressively throughout pregnancy, peak at term, and subsequently decline to near pre-pregnancy levels around the time of delivery [94]. These increased levels of leptin reflect the increasing energy-consuming processes of the maternal-placental-fetal unit [84,89]. An association between maternal leptinemia, pre-gestational BMI, and maternal weight at the end of pregnancy was found [95].

The fetus appears to regulate its leptin independently, even in the context of maternal obesity. Moreover, fetal leptin levels, but not maternal, were found to be associated with macrosomic infants and with fetal insulin [96,97,98,99]. Higher leptin, C peptide, and insulin levels were found in the cord blood of macrosomic infants compared to controls [98,100,101,102] and were independent of maternal levels [103]. Higher cord plasma concentrations of leptin and IGF-1 were significantly associated with greater fetal adiposity, suggesting that these hormones may function as early biomarkers of increased fetal fat deposition and subsequent metabolic risk [98]. However, children with congenital leptin gene mutations or leptin receptor gene mutations generally have normal weight at birth [104,105].

Prospective human cohort studies have linked cord blood leptin concentrations with postnatal growth [106,107], adiposity [107], neurodevelopment [108], and later cardiometabolic risk [109,110]. Deviations in leptin levels from an optimal range during critical periods of brain development may disrupt neurodevelopmental processes regulated by leptin, such as neuronal growth, synaptogenesis, and metabolic signaling, inducing various neurodevelopmental deviations [111,112].

However, the relationship between leptin and later adiposity is not entirely consistent across studies. While several cohorts have linked higher cord blood leptin concentrations with greater fetal adiposity and increased cardiometabolic risk later in life [98,106,107,109,110], other longitudinal studies have reported inverse or age-dependent associations. In the Project Viva cohort, higher maternal and cord blood leptin concentrations were associated with lower offspring adiposity from childhood through early adolescence, independent of maternal BMI and gestational weight gain [113]. Lower cord blood leptin concentrations were associated with smaller birth size but accelerated weight gain during infancy and higher BMI at 3 years of age [114]. Consistent with these findings, a meta-analysis of 22 studies concluded that cord blood leptin was strongly and positively associated with adiposity at birth, inversely associated with adiposity during the first 3 years of life, and not significantly associated with adiposity at 5 years of age [115]. Furthermore, the INFAT study reported an inverse association between cord blood leptin and fat mass at 2 and 3 years of age [116,117]. In contrast, data from the ALSPAC cohort suggested a modest positive association between cord blood leptin and DXA-derived fat mass at 9 years of age, although this relationship was substantially attenuated after adjustment for pregnancy-related factors [118]. Collectively, these findings suggest that leptin may function less as a direct predictor of future obesity and more as a marker of fetal energy balance and developmental adaptation. Differences in study design, age at follow-up, assessment of adiposity, fetal sex, maternal metabolic status, and adjustment for confounding variables may account for the heterogeneity observed across studies.

### 4.2. Adiponectin

In contrast to leptin, adiponectin is inversely related to leptin blood concentrations and is reduced in obese people [119]. Generally, adiponectin, a 30 kDa protein composed of 248 amino acids, is exclusively produced by adipose tissue [120]. Maternal blood adiponectin levels decrease progressively during pregnancy, particularly in the third trimester. This decline is closely associated with increased maternal fat mass and reduced insulin sensitivity. A low first-trimester adiponectin seems to be associated with increased insulin resistance and a higher risk of GDM [121,122,123].

Clinical studies indicate that maternal adiponectin concentrations are associated with fetal growth, adiposity, and size at birth. Lower early mid-pregnancy adiponectin was inversely related to infant birth weight and increased the likelihood of delivering a macrosomic infant [124]. Fetal adiponectin levels are linked to postnatal growth patterns [106,125], body composition [106], and later-life cardiometabolic [126] and neurodevelopmental outcomes, including improved cognitive performance, and better working memory [127,128].

Despite accepted evidence that blood adiponectin is associated with fetal growth and adiposity at birth, evidence regarding its relationship with later adiposity is less consistent, as different investigators reported positive, negative, or no associations of adiponectin blood levels and postnatal growth [113,121]. A meta-analysis of prospective studies found that cord blood adiponectin showed a weak positive association with adiposity at birth, whereas evidence for associations later in childhood was limited and inconsistent [115]. Longitudinal studies have shown that lower adiponectin concentrations during early life are associated with greater increases in BMI z-scores from infancy through 8 years of age, while higher leptin concentrations predict higher BMI z-scores during the same period [129]. Adiponectin and leptin concentrations measured during adolescence, as well as the adiponectin-to-leptin ratio, were strongly associated with adolescent adiposity and cardiometabolic risk, independent of adipokine levels measured at birth [126].

These discrepancies may reflect differences in adiponectin isoforms measured, particularly total versus high-molecular-weight adiponectin, age at follow-up, ethnic background, maternal metabolic status, postnatal nutrition, and statistical adjustment for birth weight and childhood growth patterns. Thus, adiponectin should not be viewed as a uniform protective factor or a simple predictor of childhood adiposity.

### 4.3. Human Placental Insulin Growth Factors (IGF) Axis

IGF-1 is a polypeptide hormone structurally related to insulin, whose biological activity is regulated by several insulin-like growth factor binding proteins (IGFBPs). During normal pregnancy, the regulation of the maternal growth hormone–insulin-like growth factor (GH-IGF) axis undergoes a physiological shift [38,39,130]. As pregnancy progresses, the placenta increasingly contributes to endocrine regulation by continuously secreting placental growth hormone (PGH) into maternal circulation. From around 15–20 weeks of gestation, PGH progressively replaces pituitary GH in the maternal bloodstream and becomes the predominant circulating GH form until delivery, while pituitary GH levels decline to near undetectable concentrations [38,131]. Through this transition, PGH becomes the primary regulator of the maternal GH-IGF axis, stimulating hepatic production of maternal IGF-1, and subsequent gluconeogenesis, lipolysis, and anabolism in maternal tissues, increasing nutrient availability for fetal growth [132,133]. Therefore, PGH promotes maternal insulin resistance, enhances lipolysis, and increases circulating glucose and lipid availability, facilitating placental nutrient transfer to the fetus.

Clinical studies support the importance of the PGH–IGF axis in determining fetal growth outcomes, especially in the second and third trimesters of pregnancy [39,40]. In addition, maternal obesity is frequently accompanied by hyperinsulinemia and increased insulin resistance, expanded pancreatic beta-cell mass, and higher insulin secretion, further stimulating placental nutrient transport, interacting with the IGF signaling pathway to promote fetal tissue growth [38].

Evidence linking maternal obesity, PGH–IGF signaling, and offspring growth is complex and appears to vary across developmental stages. Several studies have reported that maternal obesity is associated with increased maternal and fetal IGF-1 concentrations, altered IGFBP profiles, and enhanced fetal growth signals [134,135]. Maternal IGF-1 concentrations were positively associated with maternal body weight and BMI during pregnancy [136], while elevated cord blood IGF-1 concentrations have been linked to increased birth weight and neonatal adiposity, particularly among offspring of obese mothers [137]. Similarly, altered PGH–IGF–IGFBP signaling has been implicated in pregnancies characterized by fetal overgrowth and macrosomia, including those complicated by maternal obesity and diabetes [138]. Offspring birth size was also associated with IGF-binding proteins, highlighting the importance of IGF bioavailability in addition to total circulating IGF-1 levels [139].

The PGH–IGF axis likely contributes to fetal growth programming, and its effects depend on the dynamic interaction between maternal endocrine signals, placental transport capacity, and fetal nutrient demand. However, there is inadequate data to demonstrate the continuous contribution of the PGH-IGF axis postnatally.

Table 1 summarizes the hormonal imbalances in pregnancy leading to insulin resistance and postnatal overweight and obesity.

Table 1 is a summary showing how maternal conditions, including diabetes, obesity, undernutrition, and overnutrition, affect key hormonal pathways in pregnancy and are associated with fetal development and long-term metabolic outcomes. Maternal hormonal alterations, including increased insulin, leptin, insulin-like growth factor 1 (IGF-1), and cortisol, together with reduced adiponectin, are associated with systemic hormonal imbalance during pregnancy. These changes are further integrated at the placental level through modulation of the placental PGH–IGF1 axis, influencing nutrient transfer (glucose, lipids, and amino acids) and altered endocrine signaling to the fetus. At the fetal level, these signals are associated with metabolic programming, including increased lipogenesis, adipogenesis, energy storage, and hyperinsulinemia, as well as altered leptin signaling. These intrauterine adaptations are linked to variations in fetal growth, including increased adiposity, macrosomia, or compensatory catch-up growth, and may be associated with an increased risk of childhood overweight, obesity, insulin resistance, and cardiometabolic dysfunction.

These hormonal pathways provide important functional links between maternal obesity, diabetes, nutritional status, and the developmental programming of offspring health. However, evidence regarding their long-term effects is not entirely consistent, with associations often varying according to developmental stage, fetal sex, maternal metabolic status, and the timing and biological compartment of hormone assessment. Moreover, most available data are derived from observational studies and therefore remain susceptible to residual confounding by genetic, environmental, lifestyle, and postnatal factors.

**In summary**, leptin, adiponectin, and the placental GH–IGF axis hormonal systems function as key mediators linking maternal metabolic state to fetal development. Leptin reflects maternal adiposity and insulin resistance, while fetal leptin levels correlate more strongly with postnatal adiposity and metabolic risk. Adiponectin, in contrast, is inversely related to insulin resistance and modulates mainly fetal growth patterns. The GH–IGF axis further integrates maternal and placental signals to regulate nutrient availability and fetal growth trajectories.

## 5. In Utero and Early Postnatal Epigenetic Changes as Mechanisms Influencing Postnatal Weight Gain

A growing body of evidence demonstrates that diverse maternal exposures during pregnancy, including nutritional imbalance, maternal obesity, diabetes, endocrine-disrupting chemicals (EDCs), and tobacco smoke, may induce epigenetic changes in the developing embryo and fetus. These changes, which include alterations in DNA methylation, histone acetylation, chromatin structure, and gene regulatory networks, have been associated with long-term alterations in neurodevelopmental, metabolic, and immunologic pathways.

### 5.1. Nutritional Imbalance in Pregnancy

Maternal undernutrition and overnutrition are capable of reshaping fetal epigenetic programming. Both forms of nutritional stress can modify placental nutrient-signaling pathways, elevate fetal insulin, and influence fetal growth patterns [6,140,141,142]. These findings align with the DOHaD framework and are consistent with Barker’s hypothesis regarding the long-term metabolic consequences of early-life nutrient deprivation [141]. 

Evidence from famine-related undernutrition highlights the sensitivity of the epigenome to early gestational exposures. Periconceptional exposure during the Dutch Hunger Winter resulted in persistent hypomethylation of the IGF2 DMR detectable in adulthood, as shown by Heijmans et al. [143] and confirmed by Tobi et al. [144]. In contrast, early-life exposure to the Chinese Famine was associated with IGF2 hypermethylation and elevated adult cholesterol levels [145]. These contrasting findings underscore the importance of exposure timing and context, and they caution against assuming a uniform epigenetic response across cohorts.

Maternal overnutrition, including excessive gestational weight gain (EGWG, >16 kg), has been associated with distinct epigenetic alterations. The Araraquara cohort study identified 46 differentially methylated positions (DMPs) and 11 differentially methylated regions (DMRs) linked to EGWG, with enrichment in pathways related to insulin resistance and hyperglycemia [142]. These epigenetic changes were correlated with increased neonatal adiposity, suggesting that maternal metabolic status can influence early adipogenic programming.

In contrast, gestational diabetes has been associated with more extensive placental DNA methylation changes, with 256 differentially methylated positions across 133 genes identified in a cohort of 30 GDM-control pairs [146]. Some of these alterations were correlated with fetal growth parameters, particularly birth weight. However, no consistent associations were observed with neonatal metabolic biomarkers, including insulin, C-peptide, proinsulin, IGF-I, IGF-II, leptin, and adiponectin in cord blood after correction for multiple testing, suggesting that the functional impact of methylation differences may be modest or context-dependent [146]. The observed effect sizes are typically modest, often ranging from 1 to 5% DNA methylation differences, and their functional significance in early metabolic programming remains uncertain.

Most evidence linking maternal nutrition to DNA methylation and metabolic outcomes is associative, not causal, and may reflect confounding by maternal adiposity, smoking, parity, socioeconomic position, diet quality, or shared familial factors [140,141,142,147,148]. Therefore, the underlying causal pathways remain incompletely defined.

### 5.2. Endocrine Disruptors (EDCs), Fetal Growth, and Epigenetic Changes

EDCs, including phthalates, phenols, organochlorines, and PAHs, have been implicated in abnormal fetal metabolic programming. Lv et al. [145] reported that first-trimester exposure to BP-3, BPS, MEP, and triclosan was associated with higher BMI z-scores at two years of age, with CpG sites in genes such as DUXA, TMEM132C, and SEC13 accounting for a substantial proportion of this relationship [145]. In the Infancia y Medio Ambiente (INMA) cohort, persistent organochlorines (HCB, β-HCH, PCB 138, PCB 180, DDE) were strongly associated with increased BMI z-scores and overweight risk at age seven [149]. Although this analysis did not include epigenetic data, similar pollutants have been linked to methylation changes in adipogenesis and insulin-signaling genes. Urinary PAH metabolites during late pregnancy were associated with increased risk of overweight in 4- to 6-year-old children [150]. PAHs can alter methylation of CYP1A1, AHRR, and other metabolic genes in cord blood and placenta [150].

Although multiple prospective cohorts report associations between prenatal exposure to EDCs, altered DNA methylation, and subsequent child growth trajectories, the evidence remains largely associative rather than causal. Multi-pollutant analyses also associate prenatal chemical mixtures with later childhood weight outcomes [145], and epigenome-wide studies suggest that altered placental methylation patterns are related to fetal growth and downstream metabolic biomarkers [147]. Nonetheless, co-exposure to complex mixtures, exposure misclassification, and clustering with smoking, diet, and socioeconomic disadvantage complicate causal interpretation. Current human evidence links prenatal EDC exposure with adiposity-related epigenetic variation but does not establish DNA methylation as the primary causal mechanism [145].

### 5.3. Epigenetic Effects of Maternal Diet on Postnatal Growth

Maternal diet during pregnancy is increasingly recognized as a determinant of fetal epigenetic programming, with evidence from cohort and experimental studies indicating that nutritional exposures can influence DNA methylation in pathways related to growth and metabolic disease [151,152,153]. Randomized data provide stronger support for causal interpretation; for example, the PREMEDI trial reported reduced childhood overweight alongside altered methylation of leptin-related genes following Mediterranean diet intervention [154]. Similarly, epigenome-wide studies link maternal diet and metabolic status to differential methylation of key genes such as LEP and ADIPOQ, with corresponding changes in adipokine levels and fetal growth [147]. Specific dietary components, including carbohydrate intake, are associated with placental leptin methylation, supporting biologically plausible pathways linking maternal nutrition to fetal metabolic signaling [155,156].

However, the evidence remains inconsistent and methodologically limited, with some intervention studies reporting epigenetic changes while others find no significant effects [157,158]. Interpretation is also constrained by tissue specificity, because most studies rely on cord blood or placenta rather than metabolically relevant tissues such as liver, adipose tissue, muscle, or pancreas [159,160]. Thus, even when diet-related epigenetic effects are detectable, their long-term metabolic significance remains uncertain.

### 5.4. Epigenetic Effects of Early Infant Nutrition on Growth in Childhood

Early nutritional exposure may influence later risks of childhood overweight, obesity, insulin resistance, and type 2 diabetes. Breastfeeding is generally considered the preferred source of infant nutrition, whereas formula feeding has raised concerns about altered epigenetic programming during a highly plastic developmental window [161,162]. Some studies report lower DNA methylation in formula-fed infants, and breast milk may provide epigenetic signals through exosomal microRNAs and other bioactive factors that regulate genes such as FTO, INS, and IGF1 [163].

Melnik et al. proposed that human milk supports Wnt/β-catenin/TCF7L2 signaling, whereas formula feeding, particularly when high in protein and branched-chain amino acids, may favor insulin, IGF1, and mTORC1/S6K1 activation and downstream epigenetic repression of Wnt target genes [164]. This model suggests possible effects on intestinal stem cells, enteroendocrine differentiation, GLP-1 production, and pancreatic β-cell expansion [164]. Nevertheless, direct evidence for these mechanisms in humans remains limited, as most supporting data are derived from experimental models rather than studies demonstrating causal molecular changes in human tissues. Current evidence suggests that early postnatal feeding influences metabolic programming through epigenetic and signaling pathways. Although the precise mechanisms and their long-term consequences remain under investigation, early nutrition is increasingly recognized as an important contributor to lifelong metabolic health.

### 5.5. Epigenetic and Metabolic Effects of Maternal Smoking

A meta-analysis of 28 cohorts (*n* = 229,158 births) found that smoking during the first trimester increased the risk of overweight in mid-childhood (OR ≈ 1.17), with stronger effects for continued smoking during pregnancy (OR ≈ 1.42) [165]. Paternal smoking also increased risk, suggesting shared environmental exposures. Further, this study shows that exposure to prenatal smoke induces persistent methylation changes in AHRR, GFI1, and related loci, which may mediate altered metabolic programming [165]. Tobacco-related methylation changes are among the most robustly replicated epigenetic findings across human cohorts. These findings support the hypothesis that prenatal tobacco exposure contributes to metabolic programming through persistent epigenetic alterations, although the precise contribution of these changes to later metabolic dysfunction requires further investigation [160].

**Figure 1:**
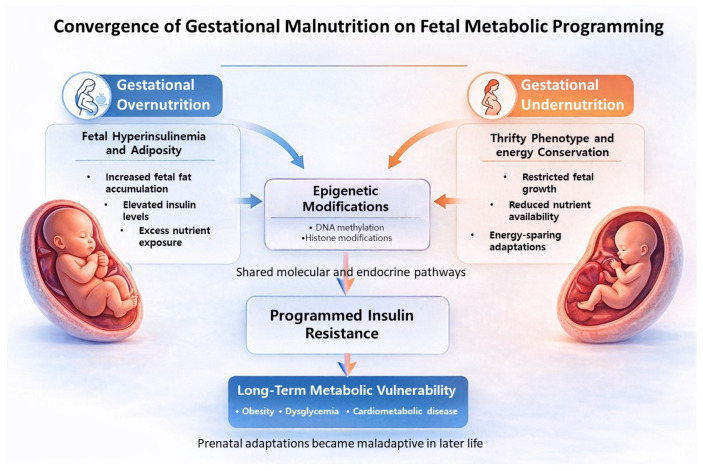
Integrated model of fetal metabolic programming related to maternal nutritional imbalance.

The Figure 1 illustrates how distinct forms of gestational malnutrition [overnutrition (left) and undernutrition (right)] ultimately converge on a shared pathway leading to fetal metabolic syndrome. Gestational overnutrition initiates fetal hyperinsulinemia and increased adiposity, while gestational undernutrition induces a thrifty, energy-conserving phenotype. Both conditions converge through shared mechanisms that include epigenetic modifications, endocrine dysregulation, and altered nutrient signaling. These processes promote programmed insulin resistance in utero, culminating in a unified fetal metabolic syndrome phenotype. However, this figure represents a simplified conceptual model and does not capture the full complexity, variability, and multifactorial nature of developmental metabolic programming.

### 5.6. Integrative Perspective: Fetal Metabolic Syndrome and Insulin Resistance with Emphasis on Gestational Undernutrition and Overnutrition

The DOHaD hypothesis proposes that the intrauterine environment programs long-term metabolic outcomes, including insulin resistance and later susceptibility to metabolic syndrome. Central to this framework is the “thrifty phenotype,” which describes adaptive metabolic responses to early-life undernutrition that become maladaptive when followed by postnatal nutritional abundance [18,74,75,76]. In contrast, gestational overnutrition, characterized as excess maternal nutrient intake that produces “excess nutrient exposure in utero,” disrupts fetal metabolic pathways and increases long-term cardiometabolic susceptibility [166]. As stated above, both undernutrition and overnutrition influence fetal development through distinct but converging pathways, representing a U-shaped pattern of risk. Undernutrition is associated with reduced insulin secretion and altered IGF signaling, whereas overnutrition is linked to fetal hyperinsulinemia, increased adiposity, and dysregulated leptin–adiponectin signaling [29,34,137]. Despite these differences, both conditions converge on insulin resistance, suggesting a shared metabolic trajectory rather than a single linear mechanism [167].

The data on nutritional imbalance during pregnancy are mainly derived from observational studies, which are associated with epigenetic and metabolomic signatures that may reflect altered growth, endocrine signaling, and intergenerational metabolic imprinting [168]. Analytical approaches such as negative controls, mediation analyses, sibling studies, and Mendelian randomization have the potential to strengthen causal inference, although they have been applied only in a limited number of studies [169]. In addition, placenta and cord blood are informative surrogate tissues, but they may not mirror organ-specific epigenetic regulation in liver, adipose tissue, skeletal muscle, or pancreas, which are central to insulin resistance and metabolic syndrome. Therefore, the extent to which DNA methylation is causal, consequential, or merely correlative remains unresolved.

Prenatal nutritional imbalance appears to shape later metabolic risk through partly overlapping pathways involving growth, endocrine signaling, and epigenetic regulation, but the strength of evidence varies substantially by exposure, tissue, and analytic design [170,171]. The ultimate outcome is also affected by postnatal nutrition, ethnicity, sociodemographic and economic factors, and the health status of the child, all of which may modify or obscure prenatal effects [172]. Thus, while the DOHaD framework remains useful, the independent impact of prenatal nutritional imbalance on long-term metabolic risk requires further investigation.

## 6. Conclusions

Many studies assess the effectiveness of different nutritional programs and diets aimed at controlling childhood obesity. They are generally only of partial success [173]. This review addresses the substantial number of children who exhibit obesity because of metabolic alterations induced by epigenetic changes originating from undesired events in pregnancy. Many of these factors are linked to permanent epigenetic changes, which may contribute to insulin resistance and increased risk of childhood and adolescent obesity, and, in adulthood, to type 2 diabetes, hypertension, and cardiovascular diseases. However, many of the conclusions in these studies are based on associations rather than causations, as they did not adequately consider possible confounding factors such as genetics, age, or the postnatal child’s nutrition (i.e., breastfeeding versus milk-based infant formula) and emotional environment. Despite these limitations, nowadays, when a large percentage of women of childbearing age are overweight, have GDM or other pregnancy-related complications, we should explore these possibilities. The EndObesity Consortium in Europe recently highlighted the first one thousand days of life, starting from conception, as the most important time for obesity prevention in children [174], recognizing pregnancy as a most important period in the etiology of childhood obesity. There is a need for well-defined studies for better delineation of the prenatal risk factors of obesity in children. There is a need for studies exploring ways to reduce the influence of maternal diseases and nutritional imbalance in pregnancy on postnatal obesity and, in addition, widespread preventive campaigns among women of childbearing age.

## Figures and Tables

**Table 1:** Maternal factors leading to offspring obesity and insulin resistance are due to hormonal imbalances.

Maternal Condition	Key Hormonal Alterations	Placental Factors	Fetal Effects	Postnatal Outcomes
**Maternal diabetes** **(T1DM, T2DM, GDM)**	↑ Glucose ↑ Insulin ↑ IGF-1 ↑ Leptin ↓ Adiponectin	↑ Placental glucose transport ↑ PGH–IGF1 axis activity Fetal pancreatic stimulation	Fetal hyperinsulinemia ↑ Lipogenesis and fat deposition ↑ anabolic signaling	Macrosomia Childhood obesity Insulin resistance Cardiometabolic risk
**Maternal overweight**	↑ Leptin (leptin resistance) ↓ Adiponectin ↑ Insulin resistance ↑ IGF-1 bioavailability	Altered placental signaling ↑ nutrient transport (glucose, lipids, amino acids) Dysregulated GH–IGF axis	Enhanced adipogenesis Altered energy balance regulation Epigenetic programming of metabolic pathways	Increased fat mass Childhood overweight/obesity Cardiometabolic risk
**Maternal undernutrition**	↓ Leptin ↓ Insulin ↑ Cortisol Adaptive “thrifty” endocrine profile	Reduced nutrient supply Altered placental growth signaling Compensatory fetal adaptations	Energy conservation programming Reduced growth → later catch-up growth Increased metabolic efficiency	Catch-up growth Central adiposity Insulin resistance Cardiometabolic risk

## Data Availability

No new data were created or analyzed in this study. Data sharing is not applicable to this article.

## Abbreviations

The following abbreviations are used in this manuscript:

BKMR	Bayesian kernel machine regression
BPA	Bisphenol A
BPS	Bisphenol S
DMP	Differentially methylated position
DMR	Differential methylation region
DOHaD	Developmental Origins of Health and Disease
EDC	Endocrine-disrupting chemical
EGWG	Excessive gestational weight gain
EPOCH	Exploring perinatal outcomes among children
FGR	Fetal growth retardation
g-comp	quantile g-computation
GDM	Gestational diabetes mellitus
GH-IGF	growth hormone–insulin-like growth factor axis
GRECF	Genomic research of the Chinese famine study
gWQS	generalized weighted quantile sum
HOMA-IR	Homeostatic model assessment of insulin resistance
hsCRP	High-sensitivity C-reactive protein
IGFBPs	Insulin-like growth factors binding proteins
INMA	Infancia y medio ambiente
LGA	Large for gestational age
MD	Mediterranean diet
PAH	Polycyclic aromatic hydrocarbons
PBDEs	Polybrominated diphenyl ethers
PGDM	Pregestational diabetes mellitus
PGH	placental growth hormone
ppBMI	pre-pregnancy body mass index
PREMEDI	Mediterranean diet during pregnancy study
SGA	Small for gestational age
